# Authenticity Assessment and Fraud Quantitation of Coffee Adulterated with Chicory, Barley, and Flours by Untargeted HPLC-UV-FLD Fingerprinting and Chemometrics

**DOI:** 10.3390/foods10040840

**Published:** 2021-04-12

**Authors:** Nerea Núñez, Javier Saurina, Oscar Núñez

**Affiliations:** 1 Department of Chemical Engineering and Analytical Chemistry, University of Barcelona, Martí i Franquès 1-11, E08028 Barcelona, Spain; xavi.saurina@ub.edu; 2 Research Institute in Food Nutrition and Food Safety, University of Barcelona, Recinte Torribera, Av. Prat de la Riba 171, Edifici de Recerca (Gaudí), Santa Coloma de Gramenet, E08921 Barcelona, Spain; 3 Serra Húnter Fellow, Generalitat de Catalunya, E08007 Barcelona, Spain

**Keywords:** coffee authenticity, HPLC-UV, HPLC-FLD, fingerprinting, chemometrics, food adulteration, chicory, barley, flours

## Abstract

Coffee, one of the most popular drinks around the world, is also one of the beverages most susceptible of being adulterated. Untargeted high-performance liquid chromatography with ultraviolet and fluorescence detection (HPLC-UV-FLD) fingerprinting strategies in combination with chemometrics were employed for the authenticity assessment and fraud quantitation of adulterated coffees involving three different and common adulterants: chicory, barley, and flours. The methodologies were applied after a solid–liquid extraction procedure with a methanol:water 50:50 (v/v) solution as extracting solvent. Chromatographic fingerprints were obtained using a Kinetex^®^ C18 reversed-phase column under gradient elution conditions using 0.1% formic acid aqueous solution and methanol as mobile phase components. The obtained coffee and adulterants extract HPLC-UV-FLD fingerprints were evaluated by partial least squares regression-discriminants analysis (PLS-DA) resulting to be excellent chemical descriptors for sample discrimination. One hundred percent classification rates for both PLS-DA calibration and prediction models were obtained. In addition, Arabica and Robusta coffee samples were adulterated with chicory, barley, and flours, and the obtained HPLC-UV-FLD fingerprints subjected to partial least squares (PLS) regression, demonstrating the feasibility of the proposed methodologies to assess coffee authenticity and to quantify adulteration levels (down to 15%), showing both calibration and prediction errors below 1.3% and 2.4%, respectively.

## 1. Introduction

Coffee, which consists of an infusion of ground roasted beans with a characteristic taste and aroma, is among the most popular drink consumed worldwide, and has become a vital product for the economic status of the countries involved in their production and exportation. The coffee plant belongs to *Coffea* genus from the Rubiaceae family, involving more than 120 species being *Canephora coffea* (Robusta) and *Arabica coffea* (Arabica), the ones with the highest economic and commercial importance [1,2,3,4]. Coffee contains a great number of bioactive substances (like phenolic acids, polyphenols, and alkaloids; with ellagic, caffeic, and chlorogenic acids among the most abundant ones) contributing to the great properties of coffee such as its antioxidant activity, well known for its beneficial health effects. In fact, some studies have related the coffee intakes with the decrease of prevalent diseases such as cirrhosis, diabetes, cancer, and cardiovascular diseases [1,5]. 

Considering coffee beneficial effects and their great popularity, the market niche becomes more competitive and, consequently, the economic cut of the coffee production ends, unfortunately in many cases, in committing adulteration frauds. Coffee adulteration is mostly performed by reducing the beans quality or by adding cheaper and lower quality coffee varieties. In addition, a growing tendency is the coffee adulteration with non-coffee materials such as corn, barley, rice, chicory, middling wheat, brown sugar, soybean, rye, stems or straw, among others, to reduce cost production and increase economic benefits [3,4,6,7,8,9]. These practices are illegal and have not only economic consequences but could also imply a danger to the consumer health. Is for these reasons that food quality control of commercial coffee products to ensure coffee authenticity and to protect the consumers is very important [6,10,11,12].Both targeted and untargeted analytical strategies have been described in the literature to address the discrimination, classification, and authentication of coffee samples based on the coffee region of production, their variety or their roasting degree. Some examples rely on liquid chromatography (LC) with ultraviolet (UV) [13,14] and fluorescence detection (FLD) [15], or LC [16], gas chromatography [17,18] and direct analysis in real-time (DART) [19] with mass spectrometry. However, in the last years, several works have been focused on the study of coffee adulteration cases either with coffees of inferior quality [14,15,20] or with different products such as chicory, corn, barley or wheat, among others [7,8,9,21,22,23,24,25]. For example, a targeted LC-UV method was employed by Song et al. for the quantification of six monosaccharides, trigonelline, and nicotinic acid for the identification of coffee powders adulterated with barley, wheat, and rice [8]. In another study, Cai et al. employed a targeted LC-mass spectrometry (MS) method to detect the presence of soybeans and rice in ground coffee by means of determining 17 oligosaccharides. Capillary electrophoresis coupled with mass spectrometry (CE-MS) has also been described as a targeted method for monosaccharide determination to detect coffee adulteration with soybean and corn [9]. 

Nowadays, untargeted fingerprinting approaches are widely employed in the literature to solve authentication problems, such as, for instance, in the case of essential oils and olive oils [26,27,28]. In the case of coffee, untargeted fingerprinting strategies based on nuclear magnetic resonance (NMR) [29], and laser induced breakdown (LIB) [7] spectroscopies, the use of electronic tongues [22], or digital images [23] have also been employed to detect and identify different coffee adulterations.

Based on the good performances previously demonstrated by untargeted high-performance liquid chromatography (HPLC)-UV and HPLC-FLD fingerprinting methodologies in the classification and authentication of coffees from different production regions and varieties [14,15,20], the present contribution aims at assessing the authenticity and the fraud quantitation on coffees adulterated with common adulterants such as chicory, barley, and different flours (wheat, rice, cornmeal, rye, and oatmeal). A simple liquid–solid extraction procedure based using methanol:water (50:50, v/v) was employed, and the C18 reversed-phase HPLC-UV-FLD fingerprints obtained from the analyzed methanolic aqueous extracts submitted to classificatory partial least squares regression-discriminants analysis (PLS-DA) chemometric methods to study their suitability as chemical descriptors for sample discrimination and authentication. Furthermore, PLS regression was employed as multivariate calibration method to detect and quantify adulterant levels on Arabica and Robusta coffees adulterated with chicory, barley, and flours.

## 2. Materials and Methods

### 2.1. Reagents and Chemicals

Methanol, ethanol, acetonitrile, and acetone (all of them Chromosolv^TM^ for HPLC, ≥99.9%) were purchased from PanReac AppliChem (Barcelona, Spain). Formic acid (≥98%) was obtained from Sigma-Aldrich (St Louis, MO, USA). Water was purified with an Elix 3 coupled to a Milli-Q system from Millipore Corporation (Millipore, Bedford, MA, USA), and was filtered through a 0.22 µm nylon membrane integrated into the Milli-Q system. 

### 2.2. Instrumentation

An Agilent 1100 Series HPLC instrument (Waldbronn, Germany) equipped with a G1312A binary pump, a WPALS G1367A automatic sample injector, a G1315B diode-array detector and a G1321A fluorescence detector connected in series, and a PC with the Agilent Chemstation software was employed to obtain the untargeted HPLC-UV and HPLC-FLD chromatographic fingerprints. Chromatographic separation was performed in a Kinetex^®^ C18 reversed-phase (100 × 4.6 mm i.d., 2.6 µm partially porous particle size) column obtained from Phenomenex (Torrance, California, USA). Gradient elution conditions using 0.1% formic acid in water (solvent A) and methanol (solvent B) as mobile phase components were employed. The elution program started increasing the methanol percentage from 3 to 75% in 30 min. Then, methanol increased from 75% to 95% in 2 min, and was kept at 95% methanol for 2 min more. After that, the elution program came back to the mobile phase initial conditions in 0.2 min and, finally, there was an isocratic step at 3% of methanol of 5.8 min to guarantee column re-equilibration. The injection volume was 5 µL and the mobile phase flow-rate was 0.4 mL/min. UV acquisition was performed at 280 nm and FLD acquisition at 310 nm (excitation) and at 410 nm (emission).

### 2.3. Samples and Sample Extraction Procedure

One hundred twenty-three samples belonging to different classes (Table 1), and purchased from supermarkets in Barcelona (Spain), Vietnam, and Cambodia, were analyzed. 

Coffee samples obtained from Vietnam were of Arabica, Robusta, and Arabica+Robusta mixture varieties. Regarding the coffee Cambodian samples, its variety was not declared in the label. Flour samples of different cereals such as wheat, rice, cornmeal, rye, and oatmeal were employed. All the analyzed samples were provided grounded by the suppliers.

Optimal sample treatment started weighing 1.00 g of sample into a 15 mL PTFE centrifuge tube (Serviquimia, Barcelona, Spain) and adding 10 mL of a methanol:water 50:50 (v/v) solution. After that, the mixture was shaken for 2 min using a Vortex (Stuart, Stone, UK). Then, the extract was centrifuged at 3500 rpm for 5 min employing a Rotanta 460 RS centrifuge (Hettich, Tuttlingen, Germany). Finally, the obtained aqueous methanolic extracts were filtered with 0.45 µm nylon filters (first mL was discarded) into an injection vial, and were stored at −4 °C until HPLC analysis. It is important to highlight that to achieve a realistic situation on coffee adulteration studies, all the barley and flour samples were submitted to a roasting process. For that purpose, 80.00 g of each sample were extended in an oven tray, and roasted for 7 min at 180 °C using a conventional oven (Teka HE 510 Me, Barcelona, Spain).

A quality control (QC) extract, prepared by mixing 50 µL of each one of the methanolic sample extracts, was used to ensure both the repeatability and robustness of the proposed methodology and the obtained chemometric results.

In addition, six coffee adulteration cases were studied involving both Vietnamese Arabica and Vietnamese Robusta coffees adulterated with chicory, barley, and wheat flour. Table 2 shows the adulteration levels (in percentage of adulterant) employed for the PLS model calibration and validation sets. An additional QC solution was also prepared at a 50% of adulterant level. For each adulteration level, five replicates were prepared, thus 55 sample extracts were analyzed for each one of the adulteration cases under study. 

### 2.4. Data Analysis

Following sample treatment, the obtained methanolic extracts were randomly analyzed with the developed HPLC-UV-FLD methods. A QC and an instrumental blank (Milli-Q water) were also injected after each ten sample extracts. Different data matrices were created with the HPLC-UV or HPLC-FLD chromatographic fingerprints of the analyzed samples. The data matrices were then analyzed by partial least squares-discriminant analysis (PLS-DA) or by partial least squares (PLS) regression methods using SOLO 8.6 chemometric software obtained from Eigenvector Research (Manson, WA, USA). Description of the theoretical background of the employed chemometric methods is addressed elsewhere [30]. In any case, the X-data matrix consisted of the acquired HPLC-UV (absorbance signal vs. retention time) or HPLC-FLD (fluorescence intensity vs. retention time) chromatographic fingerprints. Instead, Y-data matrix defined each sample classes in PLS-DA, whereas defined each adulterant percentage in PLS. Chromatographic fingerprints were normalized to achieve the same weight to each variable by suppressing differences in their magnitude and amplitude scales. PLS-DA models were also validated using 70% of the samples (randomly selected) as the calibration set and the remaining 30% of the samples as the prediction set. The most appropriate number of latent variables (LVs) in PLS-DA and PLS models were established as the first significant minimum point of the cross-validation (CV) error from a Venetian blind approach. 

## 3. Results and Discussion

### 3.1. Extraction Solvent Optimization

**Figure 1:**
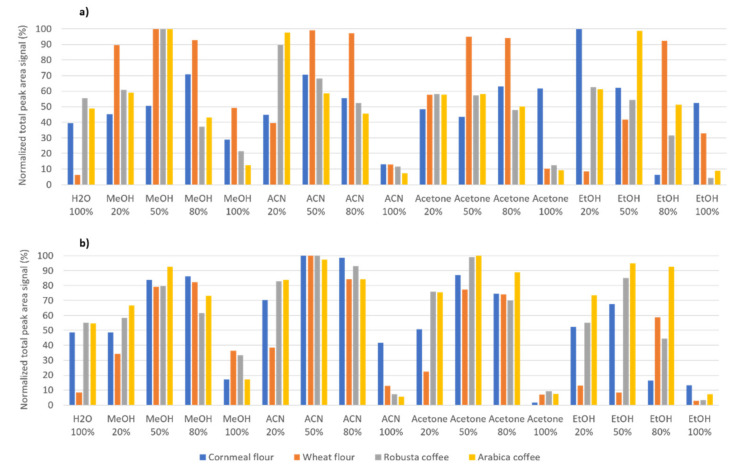
Total peak signal (normalized to the solvent providing the highest signal) of all the chemicals extracted with different extraction solvents and detected by (**a**) high-performance liquid chromatography with ultraviolet (HPLC-UV) and (**b**) HPLC- fluorescence detection (FLD) (within the chromatogram segment from 8 to 40 min) for a Vietnamese Arabica coffee, a Vietnamese Robusta coffee, a cornmeal flour, and a wheat flour.

In the present contribution, untargeted HPLC-UV and HPLC-FLD fingerprints will be exploited as sample chemical descriptors to assess coffee authenticity and to quantify adulteration levels when chicory, barley, and different flours are used as coffee adulterants. Untargeted chromatographic fingerprinting strategies are based on registering instrumental signals (in this case the absorbance and the fluorescence intensity for HPLC-UV and HPLC-FLD, respectively) as a function of the retention time, but without the requirement of any information about the chemicals present in the samples, but trying to register as much instrumental discriminant signals as possible. For that purpose, simple and generic sample treatment procedures are typically applied to extract the highest number of bioactive compounds possible and belonging to different families; although, their identification or quantification is not required. With this aim, a simple liquid–solid extraction procedure was employed, and the extraction solvent composition was optimized. Different solvents such as pure water, methanol, acetonitrile, ethanol, and acetone, and the organic aqueous mixtures containing 20%, 50%, and 80% of each organic component under study (methanol, acetonitrile, ethanol, and acetone), were evaluated as extraction solvents. Four samples, a Vietnamese Arabica coffee, a Vietnamese Robusta coffee, a cornmeal flour, and a wheat flour were employed as test samples. One gram of each sample was extracted with 10 mL of each extraction solvent following the procedure described in Section 2.3, and the obtained extracts (17 different extracts for each sample under study) were analyzed with the proposed HPLC-UV and HPLC-FLD methodology following the procedure described in Section 2.2. Chromatograms with different signal profiling depending on the sample composition were obtained. The total signal area of the chemicals extracted and detected within the chromatographic segment from minute 8 to 40 was considered as chemical data for the solvent selection (the first segment of the chromatograms was not considered to remove the signal contribution from the solvents). Figure 1 shows the total signal area (normalized to the solvent extract providing the highest signal) obtained by (a) HPLC-UV and (b) HPLC-FLD for the different samples and extraction solvents evaluated. Noticeable differences were observed depending on the sample under study as well as the fingerprinting detection system; therefore, optimal conditions will be selected as a compromise of different factors. The first thing that can be observed is that pure organic solvents (methanol, acetonitrile, acetone or ethanol) extraction capacity seems to be lower in comparison to the use of organic aqueous extraction mixtures. In addition, and as a general trend, extraction capacity increases with the organic content up to a 50% and then it decreases. 

The highest extraction capacity for all the samples under study when fluorescence detection is employed (Figure 1b) was achieved by using water:acetonitrile (50:50 v/v) as extraction solvent, obtaining almost the same normalized total peak area signal independently on the sample typology. In contrast, when ultraviolet detection was used (Figure 1a), better results were observed with water:methanol (50:50 v/v). In addition, this same solvent also provided a high extraction capacity with fluorescence detection, with normalized peak area signals higher than 80% for all the samples under study. Therefore, as a compromise, water:methanol (50:50 v/v) was selected as the optimal extraction solvent for the proposed liquid–solid extraction procedure. In addition, this solvent composition was more compatible to the HPLC mobile phase components.

### 3.2. HPLC-UV and HPLC-FLD Fingerprints 

**Figure 2:**
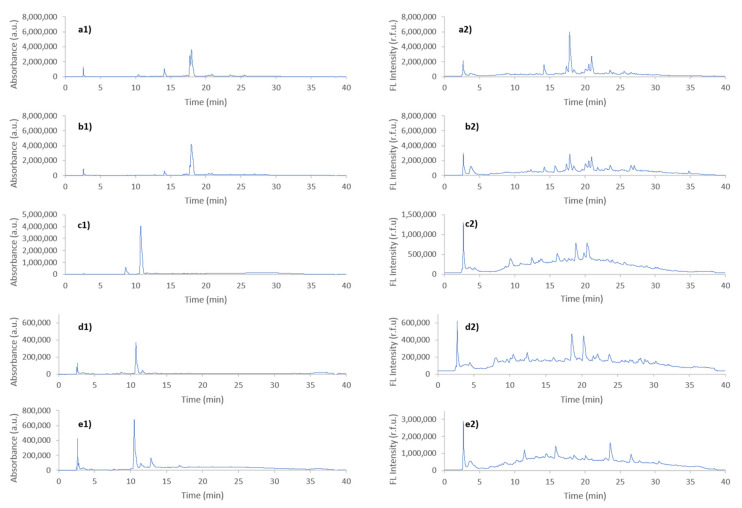
Untargeted HPLC-UV (a1–e1) and HPLC-FLD (a2–e2) fingerprints obtained for a selected sample of (**a**) Vietnamese Arabica coffee, (**b**) Vietnamese Robusta coffee, (**c**) chicory, (**d**) wheat flour, and (**e**) barley. UV detection was registered at 280 nm, and fluorescence detection at 310 nm (excitation) and 410 nm (emission).

In previous works [14,15,20], we have demonstrated that HPLC-UV and HPLC-FLD fingerprints obtained after simply brewing coffees resulted in good sample chemical descriptors to address coffee classification regarding their production region, variety, and roasting degrees. This contribution aims to assess coffee authenticity when dealing with adulterations involving the use of common non-coffee-based adulterants relying on an untargeted fingerprinting strategy. For that purpose, an important number of samples belonging to different typologies (coffee, chicory, barley, and several flours) were extracted following the sample treatment previously commented, and the obtained methanolic aqueous extracts were analyzed with the proposed HPLC-UV-FLD method. For instance, Figure 2 shows the resulting HPLC-UV (a1–e1) and HPLC-FLD (a2–e2) fingerprints for randomly selected Vietnamese Arabica coffee, Vietnamese Robusta coffee, chicory, wheat flour, and barley samples. As can be seen, important differences among the number of peak signals detected as well as their relative abundances were obtained. Regarding the number of peak signals (related to the variety of sample bioactive compounds extracted), HPLC-FLD fingerprints show less signals than the HPLC-UV ones, where very few signals are detected, although comparison regarding the total abundance cannot be done. When comparing the sample typology, it is quite clear that coffee samples provide similar fingerprints independently of the detection system employed, which are completely different to those observed for the other samples. Differences related to the coffee variety (Arabica vs. Robusta) are mainly based on relative intensities of different peak signals while following a similar fingerprinting profile. This can be clearly observed, for example, on the intensity of the peak signal detected by HPLC-FLD at minute 17 for the Vietnamese Robusta coffee (Figure 2a2) which is clearly higher in comparison to the one observed in the Vietnamese Arabia coffee sample (Figure 2b2).

As commented before, the chromatographic fingerprints obtained for the samples typically employed as coffee adulterants are completely different than those observed for coffee samples, especially regarding the peak signal intensities which tend to be much lower. However, the chicory fingerprint from UV-detection (Figure 2c1) clearly disrupt with the general fingerprinting tendency obtained for the samples considered as adulterants, showing several peaks with an important signal intensity between minutes 9 and 11 in comparison to all the other samples, including the coffee ones. Regarding fluorescence fingerprints, those obtained for barley samples seem to be richer in signals detected, as well as peak intensities, in comparison to those of chicory or wheat flour. Based on these differences, and taking into consideration that fingerprints tend to be reproducible within the same sample typology, untargeted HPLC-UV and HPLC-FLD fingerprints will be evaluated as sample chemical descriptors for the characterization and classification of the analyzed samples by chemometric analysis.

### 3.3. Sample Characterization and Classification by Chemometrics

**Figure 3:**
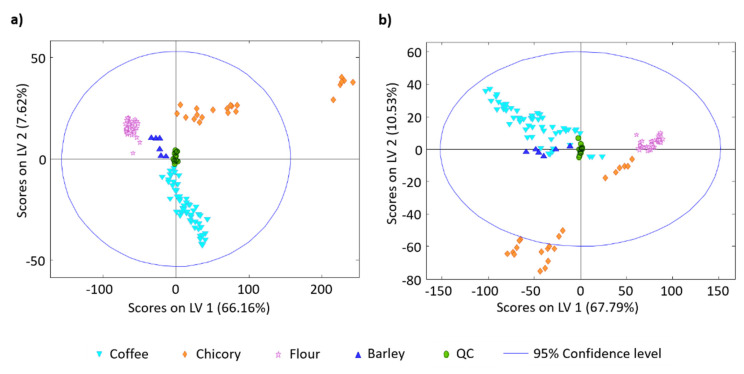
Partial least squares regression-discriminants analysis (PLS-DA) score plots of LV1 vs. LV2 for the classification of the analyzed samples when untargeted (**a**) HPLC-UV and (**b**) HPLC-FLD fingerprints were employed as sample chemical descriptors. PLS-DA models were built with 2 and 3 LVs for HPLC-UV and HPLC-FLD, respectively.

To evaluate if the obtained untargeted HPLC-UV and HPLC-FLD fingerprints worked properly as sample chemical descriptors for classification purposes, the methanolic extracts of 123 samples belonging to different typologies (see Table 1) were randomly analyzed, together with a QC sample which was injected every ten samples to evaluate both the reproducibility and the robustness of the proposed methodology and the obtained chemometric results. Then, the fingerprints were subjected to a classificatory PLS-DA chemometric method, and the resulting score plots defined by LV1 vs. LV2 are depicted in Figure 3. For that purpose, all the UV absorbance or the FL intensity signals, depending on the case, registered as a function of the chromatographic retention time, independently of the background noise observed, were used as data to build the chemometric matrices.

In both score plots, QCs appeared grouped in a compact cluster in the center area of the plot, which ensures the reproducibility of the proposed HPLC fingerprinting methodology as well as the robustness of the chemometric results. In addition, samples tend to be well grouped according to their typology, with the exception of chicory samples which form a more disperse group although perfectly discriminated from the other sample types, which may be related to the different brand and roasting process. Flour samples also appeared in quite a compacted group independently of the type or cereal (wheat, rice, cornmeal, rye, and oatmeal). Sample distribution within the score plots depends on the HPLC fingerprints used as chemical descriptors. Thus, when HPLC-UV fingerprints are employed (Figure 3a) coffee samples tend to exhibit negative LV2 values, while adulterants show positive LV2 values, and are separated from flours, barley to chicory sample with the increase in LV1 values. As a result, the four groups of samples under study are perfectly discriminated. In contrast, with HPLC-FLD fingerprints, full discrimination of all the sample groups was not accomplished. Coffee samples exhibited positive and negative LV2 and LV1 values, respectively, and are partially overlapped with barley samples; although, this last group tend to be exhibiting mainly negative LV2 values. In any case, discrimination between the three groups of adulterant samples was also accomplished, but both LV1 and LV2 are playing an important role. 

**Figure 4:**
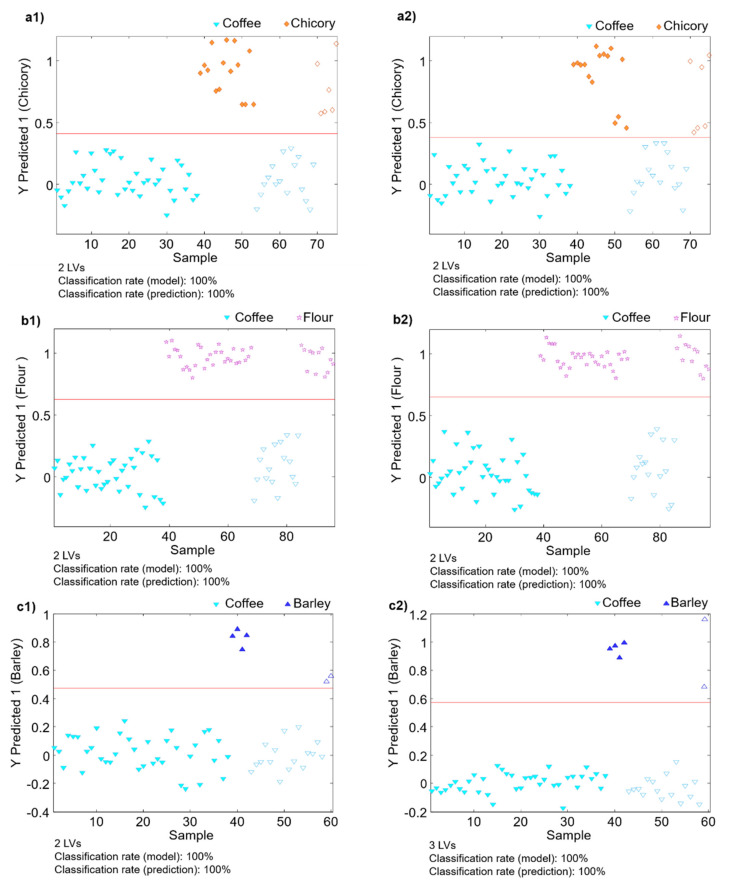
Classification plots defined by the sample vs. the predicted classes when (1) HPLC-UV (2) HPLC-FLD fingerprints were used as sample chemical descriptors. (**a**) Coffee vs. chicory samples, (**b**) coffee vs. flour samples, and (**c**) coffee vs. barley samples. Filled symbols correspond to the calibration set and empty symbols correspond to the validation set (unknown samples for prediction purposes).

As previously commented, the present contribution aims to assess coffee authenticity when adulterations with chicory, barley, or flours are taking place. For that purpose, PLS-DA models of coffee against each one of the adulterants were validated to determine the model classification rate. Thus, paired PLS-DA models were built using 70% of the samples of each group, randomly selected, as the calibration set, and the remaining 30% of samples as a validation set. They were considered as unknown samples for prediction purposes in order to evaluate the model classification performances. Figure 4 shows the obtained results for the paired PLS-DA model validations when (1) HPLC-UV and (2) HPLC-FLD fingerprints were employed as sample chemical descriptors for the classification studies of coffee against chicory (Figure 4(a1,2)), flour (Figure 4(b1,2)), and barley (Figure 4(c1,2)) adulterants. As can be seen, 100% classification rates for calibration and validation were obtained using both HPLC-UV and HPLC-FLD fingerprinting methodologies, demonstrating the feasibility of the proposed untargeted fingerprinting strategy to assess coffee classification and authentication against common non-based coffee adulterants such as chicory, barley, and flours from different cereals. 

### 3.4. Quantitation of Adulteration Levels by PLS

**Figure 5:**
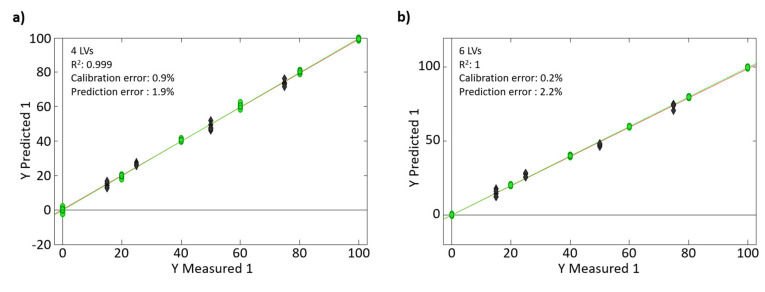
PLS regression scatter plots of measured vs. predicted percentages of adulterant for the adulteration case of Vietnamese Arabica coffee with a wheat flour when (**a**) HPLC-UV and (**b**) HPLC-FLD fingerprints were used as sample chemical descriptors.

The capacity of the untargeted HPLC-UV and HPLC-FLD fingerprinting methodologies to detect frauds and to quantify coffee adulteration levels was evaluated by PLS regression studying six adulterations cases based on both Vietnamese Arabica and Robusta coffees, each one adulterated with chicory, barley, and wheat flour, respectively. For each adulteration case under study, two independent sets of samples with different adulterant concentration levels were prepared for calibration and validation purposes, as described in Table 2. The samples were then extracted using the proposed sample treatment procedure, and the obtained methanolic aqueous extracts were randomly analyzed with the untargeted HPLC-UV-FLD method. The obtained chromatographic fingerprints were then employed as sample chemical descriptors and submitted to PLS for quantitation purposes. As an example, Figure 5 shows the scatter plots of Y measured vs. Y predicted obtained for adulteration of the Vietnamese Arabica coffee with a wheat flour when (a) HPLC-UV and (b) HPLC-FLD fingerprints were used as sample chemical descriptors.

The statistic PLS regression parameters obtained with the six adulteration cases under study and the number of LVs to build the PLS models are summarized in Table 3. As can be seen, very good results were obtained, with calibration and prediction errors always below of 1.4% and 2.4%, respectively. Both, untargeted HPLC-UV and HPLC-FLD fingerprints seem to be appropriate sample chemical descriptors for the fraud detection and quantitation, resulting in similar calibration errors (0.2–1.4% with UV and 0.2–1.3% with FLD) and prediction errors (0.9–2.2% with UV and 0.4–2.4% with FLD). 

It should be highlighted that these results are much better than those obtained when HPLC-UV and HPLC-FLD were used as sample chemical descriptors to detect and quantify coffee frauds based on adulteration with coffees of different production regions and different varieties, were calibration errors up to 3.4% and 2.9% were reported for UV and FLD, respectively, and prediction errors up to 7.5% and 18.3%, respectively [14,20]. This is probably due to the higher differences found in the chromatographic fingerprints among coffees and adulterants.

These results demonstrate the feasibility of both untargeted HPLC-UV and HPLC-FLD fingerprints of methanolic sample extracts as good sample chemical descriptors to address the detection and quantitation of adulterant levels in fraudulent coffee samples adulterated with non-based coffee adulterants such as chicory, barley, and flour.

## 4. Conclusions

Both untargeted HPLC-UV and HPLC-FLD fingerprints obtained after a sample extraction using water:methanol (50:50 v/v) have proved to be suitable sample chemical descriptors to assess the classification and authentication of coffee samples in front of common coffee adulterants such as chicory, barley, and flours. Excellent discrimination of coffee samples and the proposed adulterants was achieved by exploratory PLS-DA, especially when using HPLC-UV fingerprints. Moreover, 100% sample classification rates for both calibration and prediction were obtained when validating paired PLS-DA models of either Vietnamese Arabica or Robusta coffee against each one of the studied adulterants (chicory, barley, and flour) demonstrating the classification and authentication capacity of the proposed methodology. 

Finally, PLS multivariate calibration was applied to six adulteration cases involving a Vietnamese Robusta and a Vietnamese Arabica coffees adulterated at different levels with chicory, barley, and wheat flour, and the proposed untargeted HPLC-UV and HPLC-FLD fingerprints were appropriate to detect and quantify the adulterant levels down to 15% (lowest level evaluated for prediction) with good calibration and prediction errors (values always lower than 1.3% and 2.4%, respectively).

The proposed untargeted HPLC-UV and HPLC-FLD fingerprinting methods can be used as a simple, reliable, and relatively economic approach to assess and guarantee coffee authenticity, and to prevent fraudulent practices against adulteration with common non-coffee-based adulterants such as chicory, barley, and flours. The simplicity of an untargeted fingerprinting approach, without the requirement of using chemical standards to quantify targeted compounds, makes this methodology ideal to prevent frauds in developing coffee production countries. 

## Figures and Tables

**Table 1:** Summary of the analyzed samples.

Sample Class	Sample Type	Number of Samples
Coffee	Vietnamese Arabica coffee	13
	Vietnamese Robusta coffee	26
	Vietnamese Arabica and Robusta mixture coffee	9
	Cambodian coffee (Unknown specie)	6
Chicory	Chicory	21
Barley	Barley	6
Flour	Wheat flour	7
	Rice flour	4
	Cornmeal flour	11
	Rye flour	15
	Oatmeal flour	5

**Table 2:** Coffee and adulterant concentration levels employed for partial least squares (PLS) calibration and validation sets.

	% of Vietnamese Coffee (Arabica or Robusta)	% of Adulterant (Chicory, Barley, or Wheat Flour)
Calibration set	100	0
	80	20
	60	40
	40	60
	20	80
	0	100
Validation set	85	15
	75	25
	50	50
	25	75
	15	85

**Table 3:** PLS results for the six adulteration cases studied based on Vietnamese Arabica and Vietnamese Robusta coffees adulterated with chicory, wheat, flour, and barley.

Method	Adulterant	PLS Parameter	Vietnamese Arabica Coffee	Vietnamese Robusta Coffee
HPLC-UV fingerprinting	Chicory	LVs	5	4
		Calibration error (%)	0.2	0.6
		Prediction error (%)	1.2	0.9
	Wheat Flour	LVs	4	4
		Calibration error (%)	0.9	0.4
		Prediction error (%)	1.9	1.5
	Barley	LVs	3	3
		Calibration error (%)	1.4	1.0
		Prediction error (%)	1.5	2.2
HPLC-FLD fingerprinting	Chicory	LVs	4	3
		Calibration error (%)	0.5	0.9
		Prediction error (%)	1.1	2.0
	Wheat Flour	LVs	6	4
		Calibration error (%)	0.2	0.3
		Prediction error (%)	2.2	1.0
	Barley	LVs	4	6
		Calibration error (%)	0.4	1.3
		Prediction error (%)	0.4	2.4

## Data Availability

Data is available upon request to the authors.
